# Supplementary material for: Conditional chemoconnectomics (cCCTomics) as a strategy for efficient and conditional targeting of chemical transmission
Source: eLife. 2024 Apr 30;12:RP91927. doi: 10.7554/eLife.91927 (PMC11060718; doi:10.7554/eLife.91927)
Supplement: Figure 3—source data 3. [file elife-91927-fig3-data3.zip › Figure 3 source data3 part 2.zip!/Figure 3-Source data 3-part2/1053-1-CRISPResso_Report_mpeqg9da.zip!/CRISPResso_on_1053_3/2a.Nucleotide_percentage_quilt.pdf]

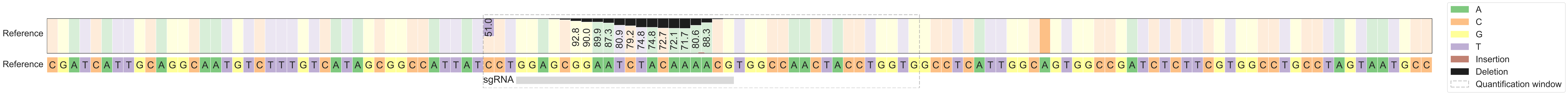
Reference
51.0
92.8
90.0
89.9
87.3
80.9
79.2
74.8
74.8
72.7
72.1
71.7
80.6
88.3
Reference
CGATCATTGCAGGCAATGTCTTTGTCATAGCGGCCATTATCCTGGAGCGGAATCTACAAAACGTGGCCAACTACCTGGTGGCCTCATTGGCAGTGGCCGATCTCTTCGTGGCCTGCCTAGTAATGCC
sgRNA
A
C
G
T
Insertion
Deletion
Quantification window
